# GLP-1 receptor signalling promotes β-cell glucose metabolism *via* mTOR-dependent HIF-1α activation

**DOI:** 10.1038/s41598-017-02838-2

**Published:** 2017-06-01

**Authors:** Rodrigo Carlessi, Younan Chen, Jordan Rowlands, Vinicius F. Cruzat, Kevin N. Keane, Lauren Egan, Cyril Mamotte, Rebecca Stokes, Jenny E. Gunton, Paulo Ivo Homem de Bittencourt, Philip Newsholme

**Affiliations:** 1 School of Biomedical Sciences, Curtin Health Innovation Research Institute, Perth, WA Australia; 2 Post-Graduate Program in Medical Sciences: Endocrinology, Federal University of Rio Grande do Sul, Porto Alegre, Brazil; 3 Key Laboratory of Transplant Engineering and Immunology, NHFPC; Regenerative Medicine Research Center, West China Hospital, Sichuan University, Chengdu, P.R. China; 4 Centre for Diabetes, Obesity and Endocrinology, The Westmead Millennium Institute for Medical Research, The University of Sydney, Westmead, NSW Australia; 5 Diabetes and Transcription Factors Group, Garvan Institute of Medical Research, NSW, Australia; 6 Department of Physiology, Institute of Basic Health Sciences, Federal University of Rio Grande do Sul, Porto Alegre, Brazil

## Abstract

Glucagon-like peptide-1 (GLP-1) promotes insulin secretion from pancreatic β-cells in a glucose dependent manner. Several pathways mediate this action by rapid, kinase phosphorylation-dependent, but gene expression-independent mechanisms. Since GLP-1-induced insulin secretion requires glucose metabolism, we aimed to address the hypothesis that GLP-1 receptor (GLP-1R) signalling can modulate glucose uptake and utilization in β-cells. We have assessed various metabolic parameters after short and long exposure of clonal BRIN-BD11 β-cells and rodent islets to the GLP-1R agonist Exendin-4 (50 nM). Here we report for the first time that prolonged stimulation of the GLP-1R for 18 hours promotes metabolic reprogramming of β-cells. This is evidenced by up-regulation of glycolytic enzyme expression, increased rates of glucose uptake and consumption, as well as augmented ATP content, insulin secretion and glycolytic flux after removal of Exendin-4. In our model, depletion of Hypoxia-Inducible Factor 1 alpha (HIF-1α) impaired the effects of Exendin-4 on glucose metabolism, while pharmacological inhibition of Phosphoinositide 3-kinase (PI3K) or mTOR completely abolished such effects. Considering the central role of glucose catabolism for stimulus-secretion coupling in β-cells, our findings suggest that chronic GLP-1 actions on insulin secretion include elevated β-cell glucose metabolism. Moreover, our data reveal novel aspects of GLP-1 stimulated insulin secretion involving *de novo* gene expression.

## Introduction

Insulin release is a complex and highly controlled process^1^. It is dependent on stimulus-secretion coupling, whereby glucose catabolism within pancreatic β-cells generates the primary signal for secretion. ATP generated through glycolysis and mitochondrial respiration provides the key signal for closure of ATP-sensitive K^+^ channels, subsequently causing membrane depolarization and activation of voltage-dependent Ca^2+^ channels (VDCCs), Ca^2+^ influx and exocytosis of insulin vesicles^2^. Therefore, the normal response to carbohydrate containing meals involves a rise in blood glucose concentration and increased β-cell glucose metabolism, subsequently promoting insulin secretion. However the latter responses are impaired in type 2 diabetes (T2D), where β-cell dysfunction plays a major role^3^. Thus, any treatment capable of augmenting glucose metabolism in β-cells may result in clinical benefit from improvement in insulin secretion and overall glucose homeostasis.

Glucagon-like peptide-1 (GLP-1) physiologically induces glucose-dependent insulin secretion from β-cells and GLP-1 analogues ameliorate hyperglycaemia in T2D patients^4–6^. GLP-1 exerts its actions by binding to a G-protein coupled receptor (GLP-1R) expressed on the surface of many cells including β-cells, which, upon stimulation, leads to rapid activation of adenylyl cyclase thus increasing cAMP levels^7^. cAMP directly activates protein kinase A (PKA) and cAMP-regulated guanine nucleotide exchange factor 2 (Epac2), that act in concert to generate downstream signals resulting in increased insulin secretion^8^. Mechanisms include ATP sensitive K^+^ channel closure, facilitation of VDCCs opening, inhibiting membrane repolarization *via* Kv channels and Ca^2+^-induced Ca^2+^ release from cytoplasmic storage sites^9–14^.

All known mechanisms of GLP-1-induced insulin secretion depend on glucose metabolism. Thus, it is attractive to hypothesize that GLP-1 signalling could enhance flux through the glycolytic pathway to generate metabolic stimulus-secretion factors. However, it has previously been reported that acute exposure to GLP-1 does not affect energy metabolism in β-cells^15^. On the other hand, prolonged (16 h) stimulation with GLP-1 was shown to promote Hypoxia-Inducible Factor 1 (HIF-1) activity *via* induction of the mammalian Target of Rapamycin (mTOR)^16^. HIF-1 is a heterodimeric transcriptional factor composed of two subunits, HIF-1α and HIF-1β^17^. It induces metabolic reprogramming in response to hypoxia and growth factor signalling^18, 19^, partly by promoting transcriptional activation of glycolytic genes^20^. This piece of evidence suggests that GLP-1 may induce late metabolic changes, not yet elucidated, downstream to HIF-1α activation.

Here we show that chronic stimulation of the GLP-1R increases glycolysis and ATP production in β-cells through transcriptional activation and expression of glycolytic genes. Pharmacological inhibition of the PI3K/mTOR pathway abolished such effects, suggesting that the metabolic actions of GLP-1 depend on mTOR activity. In addition, we observed that HIF-1α protein levels accumulate downstream of mTOR in response to GLP-1R signalling, whereas we also demonstrated that depletion of HIF-1α impaired effects on glycolysis and transcriptional regulation of glycolytic genes. We propose that chronic exposure to GLP-1 signalling promotes mTOR-dependent metabolic reprograming *via* activation of the HIF-1 transcriptional program. Such metabolic reprograming persists after removal of receptor stimulation, this was evidenced by increased levels of insulin secretion and glucose utilization following withdrawal of Exendin-4.

## Results

### Prolonged GLP-1R Signalling Stimulates Glycolysis *via* Up-Regulation of Glycolytic Enzymes

**Figure 1:**
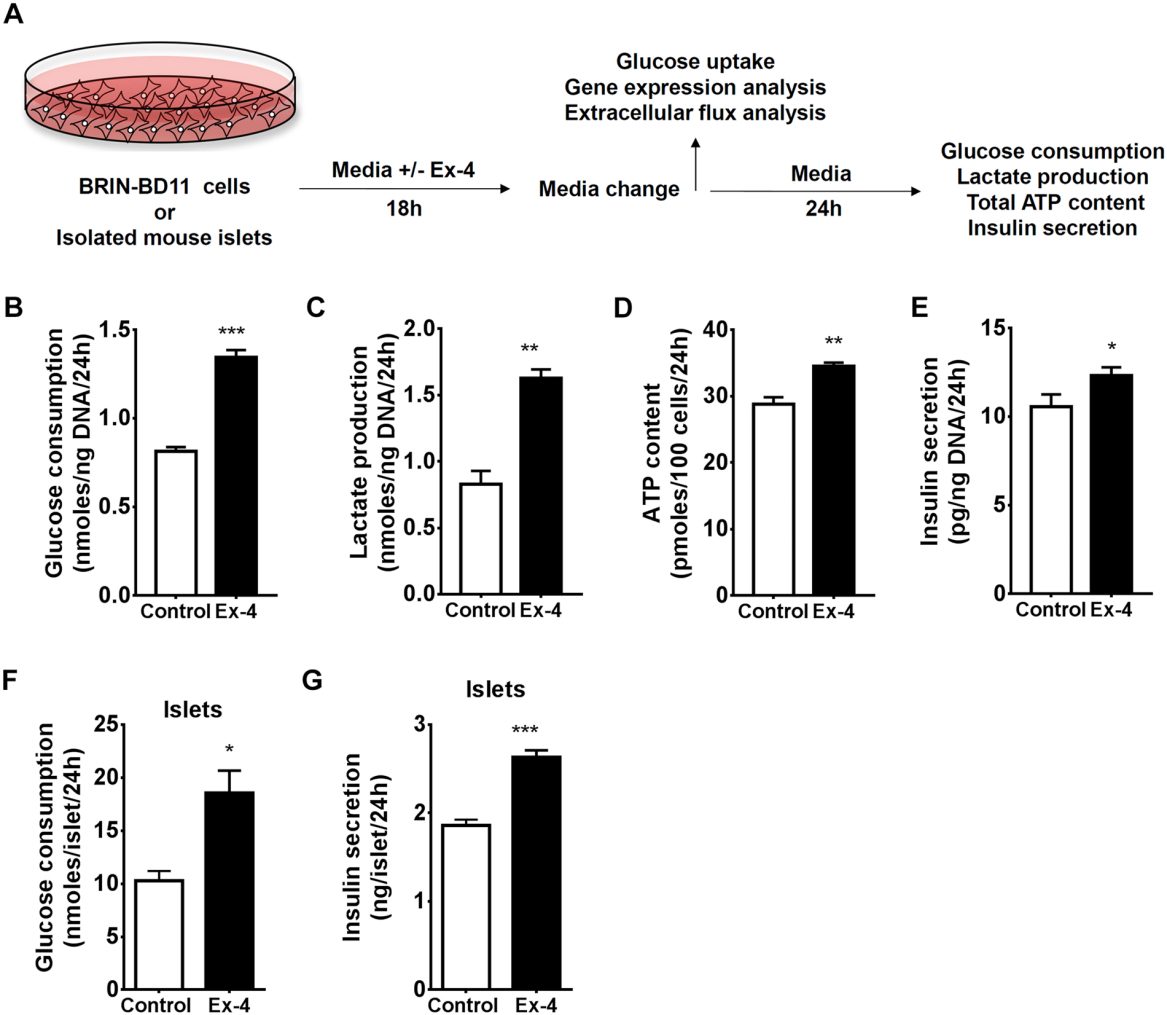
Chronic GLP-1R signalling stimulates β-cell glycolytic metabolism and function. (**A**) Schematic diagram outlining experimental procedures. (**B**) Glucose consumption, (**C**) lactate production, (**D**) ATP content, and (**E**) insulin secretion were assessed in BRIN-BD11 cells. (**F**) Glucose consumption and (**G**) insulin secretion were assessed in murine islets. Data represent mean ± SEM, n ≥ 3; *P < 0.05; **P < 0.01; ***P < 0.001.

GLP-1 requires the presence of glucose for stimulation of insulin secretion^14^; thus we investigated if GLP-1 could modulate glucose metabolism in β-cells. Rat insulin-secreting BRIN-BD11 cells or isolated murine islets were stimulated for 18 hours with 50 nM of the GLP-1 analogue Exendin-4 and a high (20 mM) glucose concentration. Media was changed and cells cultured for additional 24 hours in the absence of Exendin-4. The rationale for this approach was to investigate if GLP-1 signalling could cause metabolic reprograming that would persist after removal of receptor stimulation (Fig. 1A). Glucose consumption, lactate production, total ATP content and insulin secretion were determined after 24 hours of incubation in the absence of Exendin-4 (Fig. 1B–G). All parameters were significantly enhanced in cells pre-conditioned with Exendin-4 relative to control cells treated with high glucose alone. Exendin-4 may affect β-cell number by stimulating cellular proliferation^21, 22^. However, in our experimental model with BRIN-BD11 cells, 18 h exposure to 50 nM Ex-4 did not induce significant changes in cell numbers (Fig. S1). All primary outcomes presented herein were normalized by total DNA content, eliminating cell number effects from the interpretation of our data.

**Figure 2:**
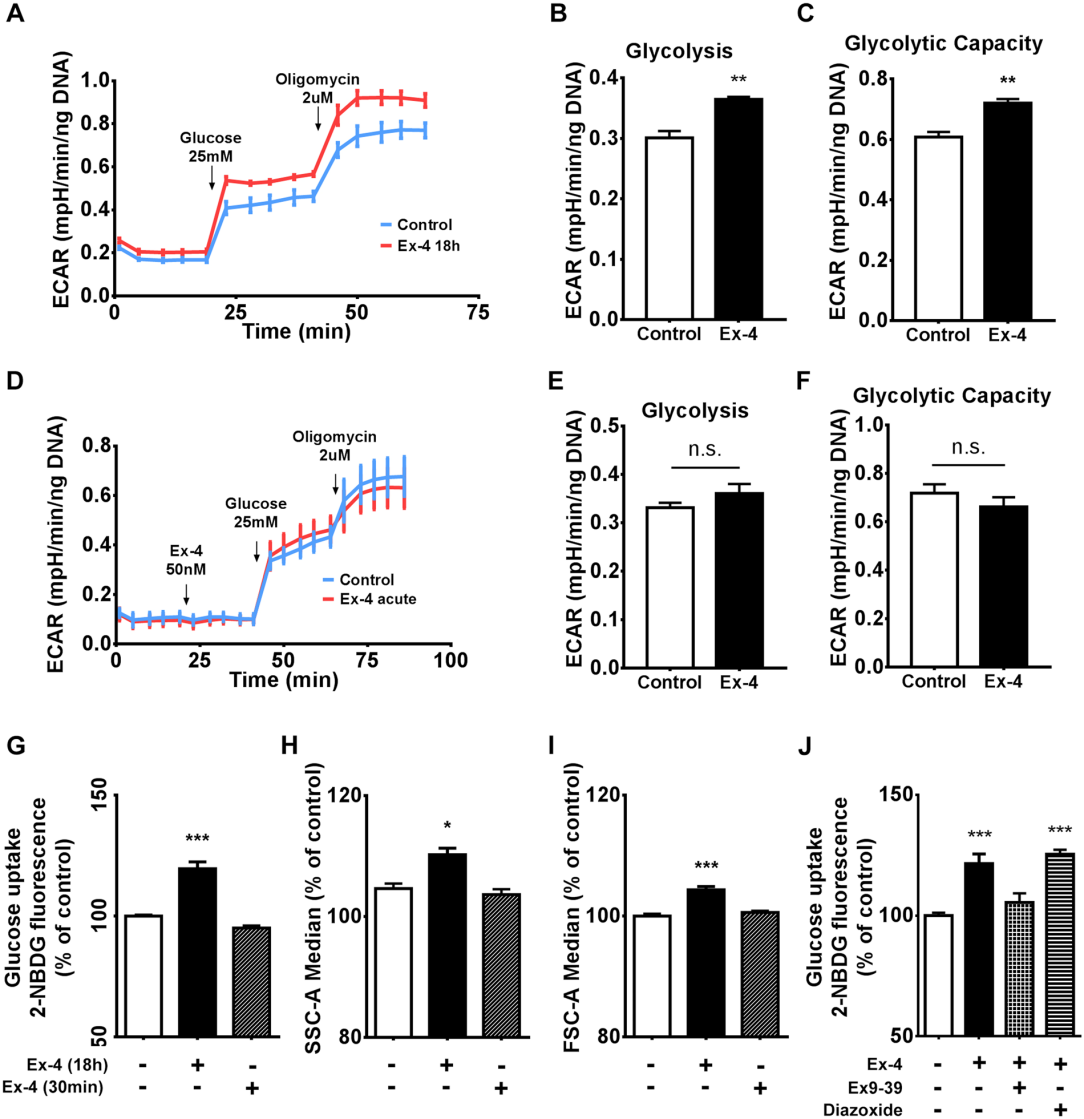
Prolonged, but not acute, Exendin-4 stimulation promotes metabolic reprogramming. Following 18 hours of incubation in the presence or absence of 50 nM Exendin-4, BRIN-BD11 cells were subjected to extracellular flux analysis. (**A**) ECAR was determined after sequential injection of 25 mM glucose and 2 µM oligomycin. (**B** and **C**) Glycolytic rate and capacity were calculated as detailed in Methods. (**D**) ECAR profiles were generated using control untreated cells to evaluate the effect of acute injection of 50 nM Exendin-4 compared to cells injected with control media, followed by 25 mM glucose and 2 µM oligomycin. (**E** and **F**) Glycolytic rate and capacity were calculated. (**G**) Uptake of 2-NBDG was determined by flow cytometry analysis after preconditioning in the presence or absence of 50 nM Exendin-4 for either 18 h or 30 min. (**H** and **I**) The same treatments were evaluated in respect to SSC-A and FSC-A to estimate cellular complexity and size respectively. (**J**) 2-NBDG uptake was determined after co-incubation for 18 h with 1 µM Exendin (9–39) or 250 µM diazoxide. Data represent mean ± SEM, n ≥ 3; n.s. = non-significant *P < 0.05; **P < 0.01; ***P < 0.001.

Next, we assessed glycolytic flux by measuring extracellular acidification rates (ECAR) using the XF^e^96 flux analyzer. Exendin-4 pre-conditioned cells responded to both glucose and oligomycin injections by increasing their ECAR levels substantially higher than control cells (Fig. 2A). Basal glycolysis and glycolytic capacity can be calculated by subtraction of ECAR values obtained after glucose and oligomycin injections from basal ECAR levels respectively. ECAR measurements indicated that basal glycolysis (Fig. 2B) and glycolytic capacity (Fig. 2C) were both increased by Exendin-4 pre-conditioning. In order to confirm that the observed glycolytic effects require prolonged exposure to GLP-1R activation, we performed flux analysis after acute injection of Exendin-4 (Fig. 2D). Calculation of basal glycolysis and glycolytic capacity rates revealed an absence of any significant change after acute injection of Exendin-4 (Fig. 2E and F). To validate these findings, we assessed glucose uptake rates through flow cytometric measurement of a fluorescently labelled glucose analogue (2-NBDG). This demonstrated that cells pre-treated with Exendin-4 for 18 h responded by an approximate 25% increase in the rate of 2-NBDG uptake, whereas exposure to Exendin-4 for only 30 minutes did not elicit any significant change (Fig. 2G). Interestingly, cellular complexity and size, as estimated by side scatter (SSC) and forward scatter (FSC) respectively, both were increased as a result of 18 h Exendin-4 pre-treatment, but not after 30 minutes of exposure (Fig. 2H and I). Altogether, this dataset suggests that prolonged, but not acute, activation of GLP-1R induces metabolic reprogramming leading to increased glycolysis and cellular hypertrophy in BRIN-BD11 cells. To confirm that the observed effects were mediated through direct GLP-1R activation, or indeed, that they are not secondary autocrine effects of insulin, we performed 2-NBDG uptake experiments in the presence of the GLP1R antagonist Exendin (9–39) and the potassium channel opener, diazoxide. The effects of Exendin-4 were abolished in the presence of Exendin (9–39), but remained intact when insulin secretion was abrogated by diazoxide (Fig. 2J). This confirms that the observed metabolic reprogramming was dependent on chronic activation of GLP-1R signalling and independent of its acute stimulation of insulin secretion, which may result in autocrine insulin signalling.

**Figure 3:**
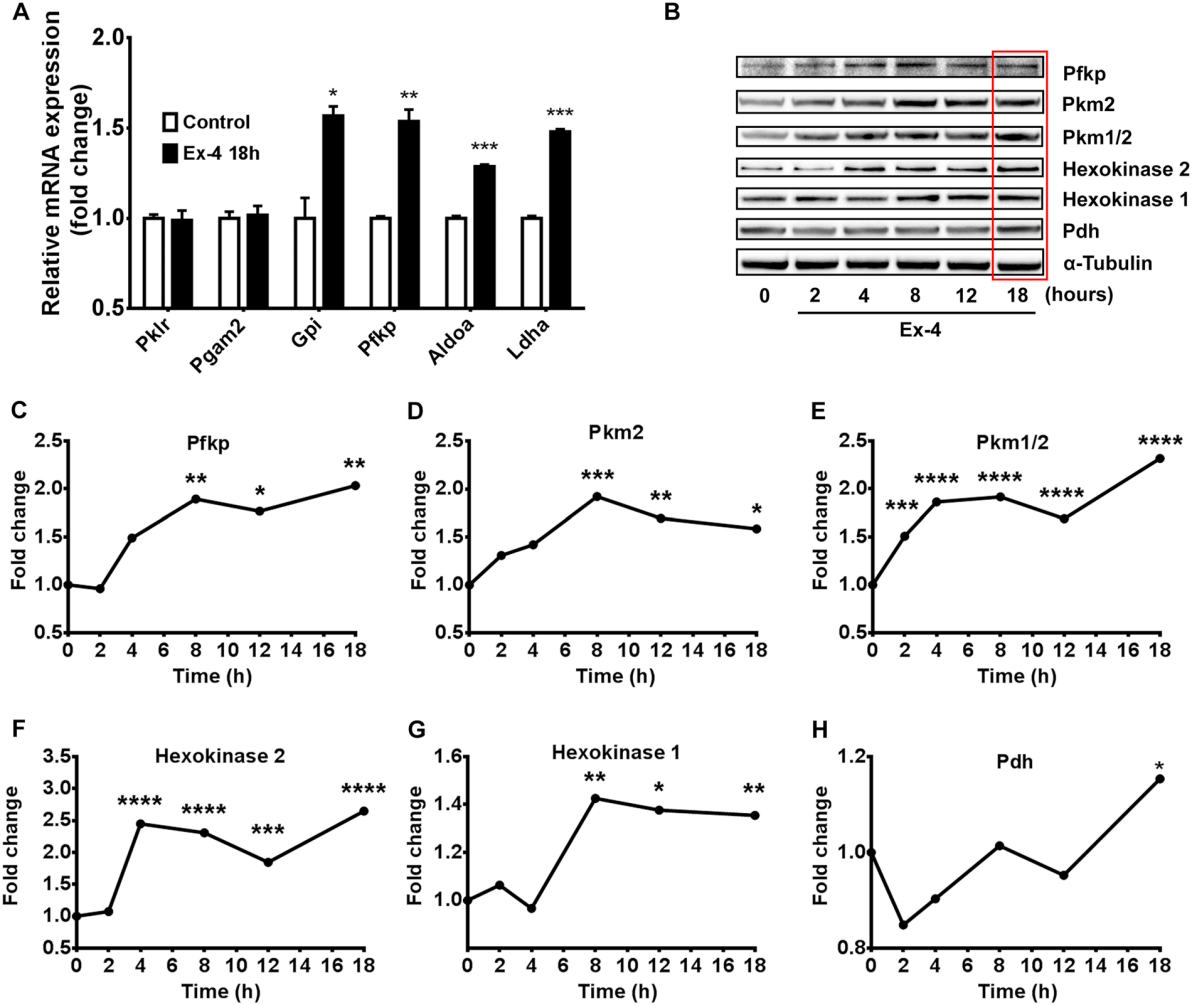
18 h of GLP-1R stimulation induces mRNA and protein expression of glycolytic enzymes. (**A**) mRNA expression of the glycolytic enzymes Pyruvate Kinase (Pklr), Lactate dehydrogenase A (Ldha), Aldolase A (Aldoa), Phosphofructokinase (Pfkp), Glucose-6-phosphate isomerase (Gpi) and Phosphoglycerate mutase 2 (Pgam2) was assessed by qRT-PCR in BRIN-BD11 cells exposed (or not) to 50 nM Exendin-4 for 18 hours. (**B**) Protein levels of glycolytic enzymes were analysed by immunoblot following 0, 2, 4, 8, 12 and 18 hours of exposure to 50 nM Exendin-4. Expression levels of (**C**) Pfkp, (**D** and **E**) Pyruvate Kinase M1 and M2 (Pkm1, Pkm2), (**F**) Hexokinase 1, (**G**) Hexokinase 2 and (**H**) Pyruvate dehydrogenase α1 subunit (Pdh) were quantified by band densitometry analysis. Data represent mean ± SEM, n ≥ 3; *P < 0.05; **P < 0.01; ***P < 0.001; ****P < 0.0001.

Flux through the glycolytic pathway is tightly controlled through regulation of glycolytic enzyme activity by allosteric modulation, post-translational modifications as well as transcriptional control. The time required for each of these regulatory mechanisms to occur is typically in milliseconds, seconds, and hours, respectively^23^. The extended time required for the effects observed here to take place suggests that they include regulation of gene expression. Hence, we assessed mRNA and protein expression profiles of genes involved in the glycolytic pathway. All genes analysed give rise to enzymes that catalyse rate-controlling steps in glycolysis, and therefore changes in their expression levels have the potential to affect the glycolytic flux. Four of the six genes investigated by quantitative RT-PCR were significantly up-regulated by Exendin-4 pre-conditioning, including Lactate dehydrogenase A (*Ldha*), Aldolase A (*Aldoa*), Phosphofructokinase (*Pfkp*) and Glucose-6-phosphate isomerase (*Gpi*) (Fig. 3A). Furthermore, we used a commercial glycolysis antibody panel to detect Pfkp, Pyruvate kinase M1 and M2 (Pkm1, Pkm2), Hexokinase 1, Hexokinase 2 and Pyruvate dehydrogenase α1 subunit (Pdh) by immunoblot analysis. A time-dependent increase in protein levels of the analysed glycolytic proteins was observed in response to Exendin-4 stimulation (Fig. 3B–H). Glucose transporter 2 (Glut2) and Glucokinase, which respectively mediate two early rate-controlling steps in the glycolytic pathway, glucose uptake and carbon 6 phosphorylation respectively were also investigated. Immunoblot analysis, however, indicated no significant changes in the protein levels of Glut2 and Glucokinase in response to Exendin-4 treatment (Fig. S2).

### Maximal Mitochondrial Respiration and Reserve Capacity are induced after Prolonged GLP-1R Activation

**Figure 4:**
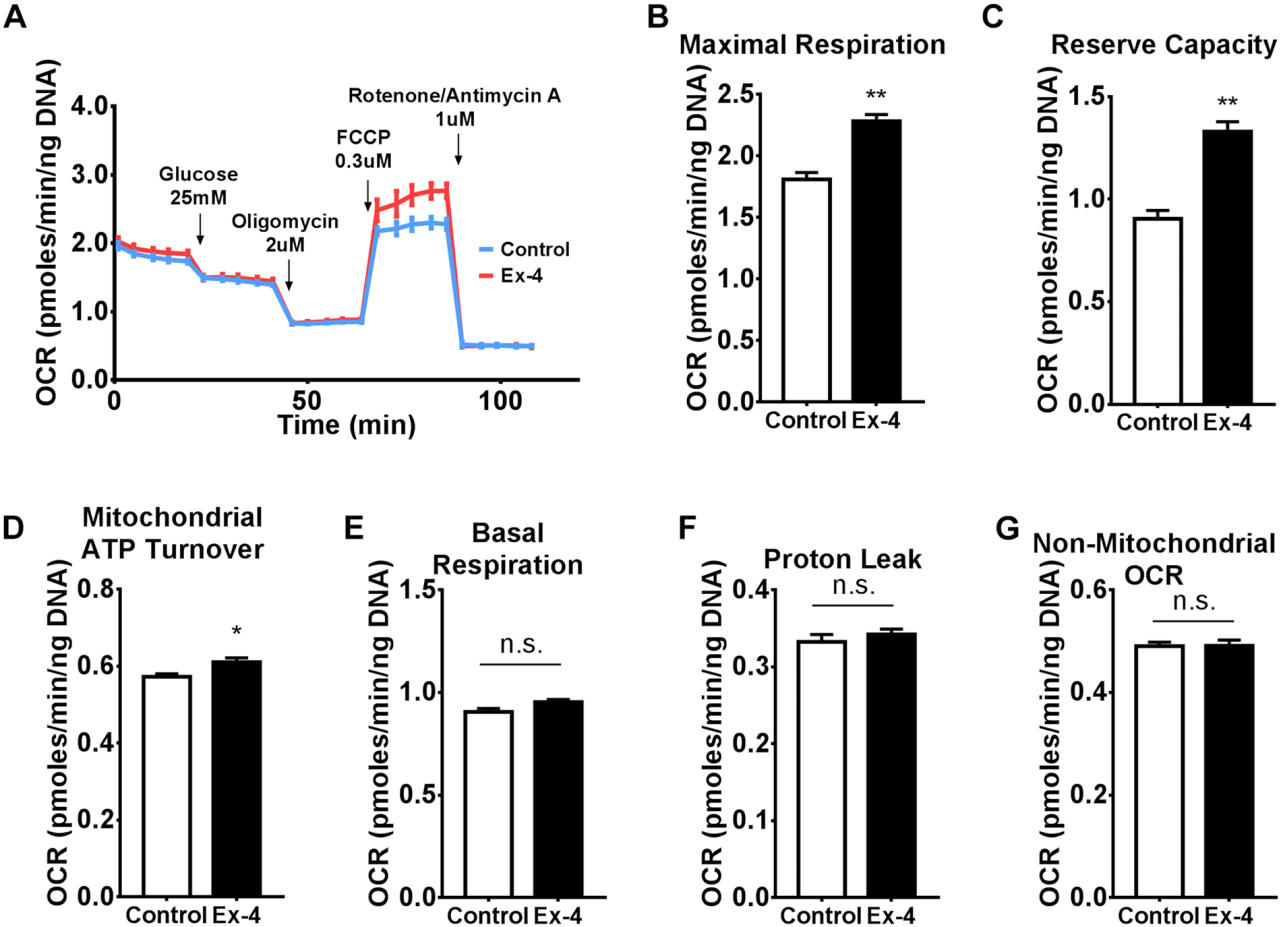
Maximal respiration and reserve capacity are increased after 18 h exposure to Exendin-4. (**A**) Following 18 h of incubation in the presence or absence of 50 nM Exendin-4, BRIN-BD11 cells were subjected to extracellular flux analysis. OCR was determined sequentially after injection of 25 mM glucose followed by a set of mitochondrial inhibitors (2 µM oligomycin, 0.3 µM FCCP and 1 µM each of rotenone and antimycin A) to generate a mitochondrial stress profile. (**B**–**G**) Mitochondrial parameters were calculated from the mitochondrial stress profile as described in Methods. (**B**) Maximal respiration, (**C**) reserve capacity, (**D**) mitochondrial ATP turnover, (**E**) basal respiration, (**F**) proton leak, (**G**) non-mitochondrial OCR. Data represent mean ± SEM, n = 3; n.s. = non-significant; *P < 0.05; **P < 0.01.

Extracellular flux analysis can be used to assess mitochondrial bioenergetics through measurements of oxygen consumption rates (OCR) following the injection of specific mitochondrial inhibitors^24^. We measured OCR in cells either pre-conditioned, or not, with Exendin-4 after injection of glucose, oligomycin, FCCP and a combination of rotenone and antimycin A (Fig. 4A). This strategy allowed us to estimate several mitochondrial parameters in response to GLP-1R stimulation. Maximal mitochondrial respiration and reserve capacity were strikingly enhanced by Exendin-4 preconditioning (Fig. 4B and C, respectively), whereas mitochondrial ATP turnover was slightly increased (Fig. 3D). On the other hand, basal respiration (Fig. 4E), proton leak (Fig. 4F) and non-mitochondrial OCR (Fig. 4G) were unaffected. Interestingly, maximal respiration and reserve capacity are both estimated from OCR measurements after injection of FCCP, an uncoupling agent that elevates mitochondria to their maximum electron transport capacity. Under these conditions, the production of glycolytic derived pyruvate becomes rate limiting for the Krebs cycle and, consequently, to mitochondrial electron transport. The observed effects in maximal respiration and reserve capacity most likely occur due to increased glycolysis and consequent augmented flux of pyruvate into the Krebs cycle.

### PI3K/mTOR Axis Mediates GLP-1R Signalling Actions on Glucose Metabolism

**Figure 5:**
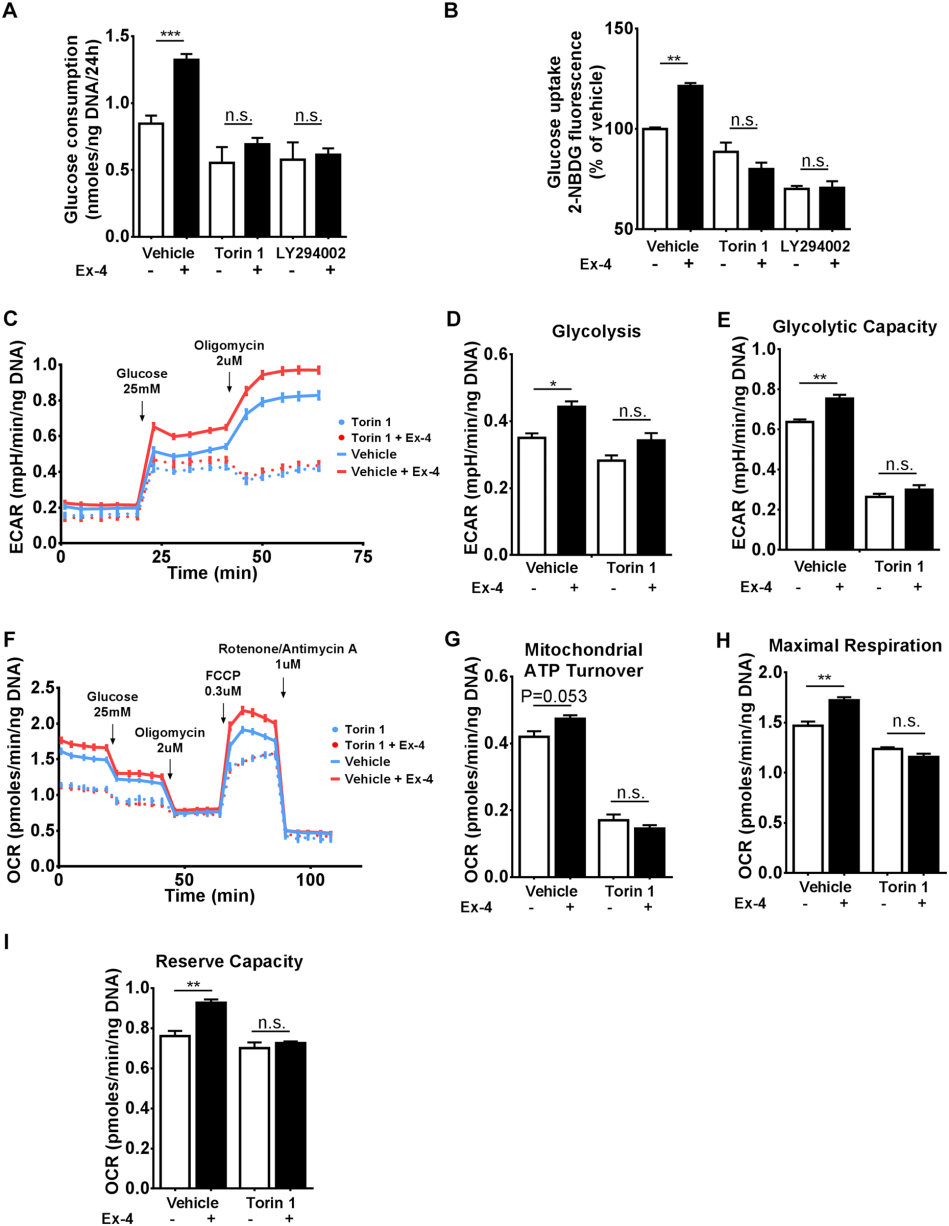
Inhibition of the PI3K/mTOR axis abolishes the effects of GLP-1R signalling on cellular metabolism. (**A**) BRIN-BD11 cells were pre-incubated with either vehicle, 1 µM Torin 1, or 50 µM LY294002 for 30 min, followed by addition (or not) of 50 nM Exendin-4 for 18 hours, as indicated. Then, media was changed to RPMI containing 20 mM of glucose for additional 24 hours in the absence of Exendin-4 and glucose consumption was determined. (**B**–**I**) Cells were treated similarly to (**A**), but instead of the additional 24 hours incubation time, 2-NBDG uptake or extracellular flux analysis were performed immediately following the 18 hours incubation period and parameters calculated as described in Methods. Data represent mean ± SEM, n ≥ 3; n.s. = non-significant; *P < 0.05; **P < 0.01; ***P < 0.001.

GLP-1 can regulate cell size, function and proliferation in β-cells *via* PI3K/mTOR pathway^25, 26^. Moreover, mTOR controls nutrient sensing and energy metabolism in various cell types^27^. Co-incubation of Exendin-4 with either LY294002 or Torin-1, two inhibitors of PI3K and mTOR respectively, completely abrogated the effect of Exendin-4 on glucose consumption (Fig. 5A) and uptake (Fig. 5B). The ECAR profile (Fig. 5C) revealed that mTOR inhibition slightly impaired responsiveness to glucose, but entirely abrogated cellular ability to increase ECAR to compensate for oligomycin-induced blockade of mitochondrial ATP production. Most importantly, Torin 1 abolished the observed increase in ECAR in response to Exendin-4. Estimation of basal glycolysis (Fig. 5D) and glycolytic capacity (Fig. 5E) indicated that inhibition of mTOR blunted such metabolic adaptations induced by GLP-1R activation.

Next, we assessed the role of mTOR in mediating Exendin-4 effects on mitochondrial bioenergetics (Fig. 5F). mTOR inhibition not only impaired mitochondrial ATP turnover, but abolished the positive effect of Exendin-4 on this parameter (Fig. 5G). In addition, the effects of Exendin-4 on maximal mitochondrial respiration (Fig. 5H) and reserve capacity (Fig. 5I) were completely blocked by inhibition of mTOR.

Torin-1 is a potent inhibitor of both mTOR complexes 1 and 2, namely mTORC1 and mTORC2. In order to investigate specifically the role of mTORC1, we determined Exendin-4 effects on glucose consumption and uptake under inhibition of mTORC1 by Rapamycin, a compound that does not inhibit mTORC2 at the concentration tested (100 nM) (Fig. S2A and B, respectively). The results of these experiments demonstrate that in spite of the role of mTORC1 in the control of energy metabolism, as evidenced by a significant reduction of overall glucose consumption and uptake following Rapamycin treatment; Exendin-4 could still exhibit stimulatory activity in the same setting, suggesting that either mTORC2 alone or both mTOR complexes must be blocked in order to completely abrogate GLP-1R signalling action on glycolysis. Our data confirm the importance of mTOR activity in the regulation of β-cell energy metabolism and provide evidence that chronic GLP-1R signalling effects are mediated by the PI3K/mTOR pathway.

### HIF-1α Accumulates Downstream of mTOR and is required for GLP-1-Induced Metabolic Reprograming

**Figure 6:**
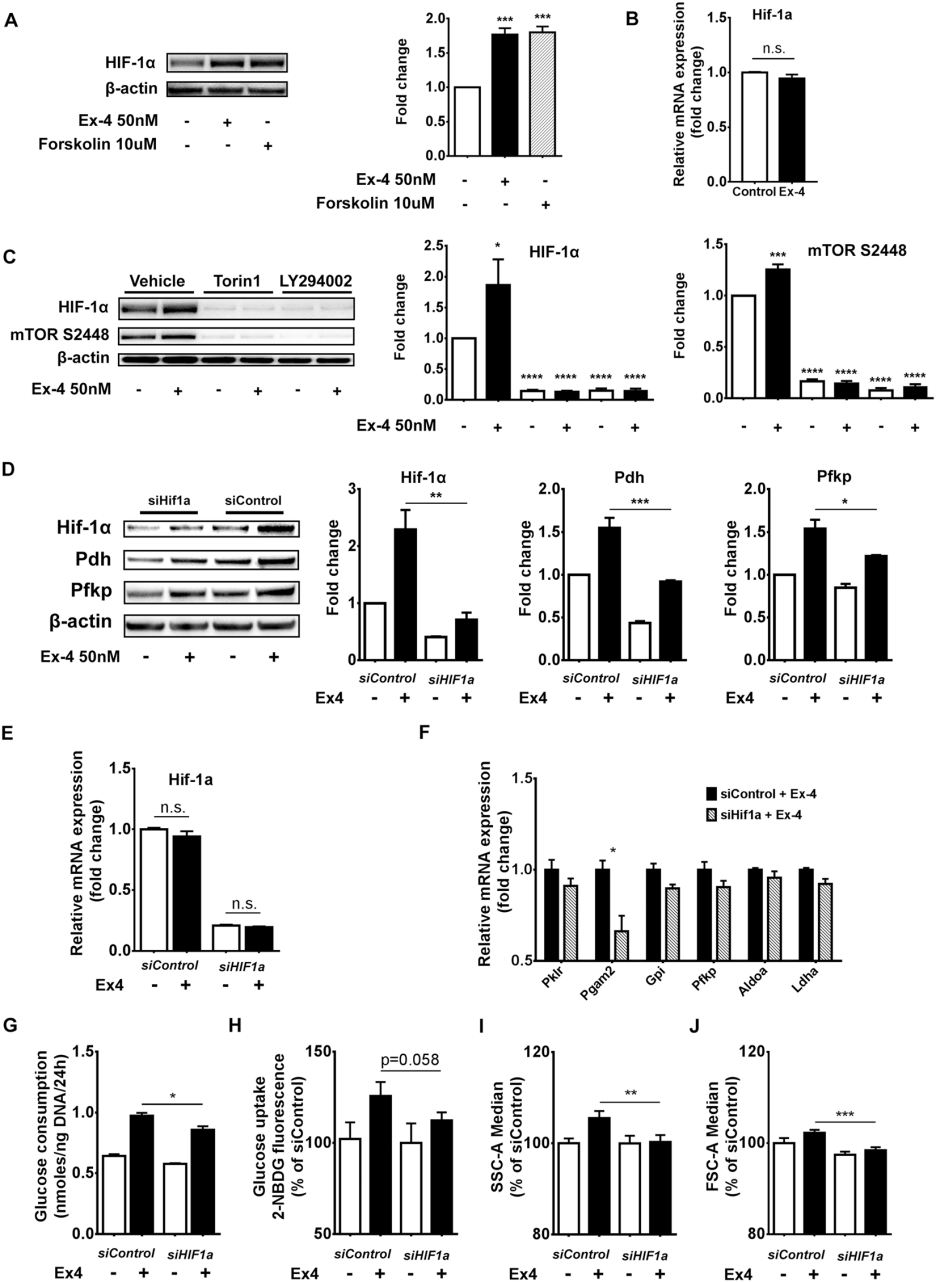
Increase in glucose metabolism induced by GLP-1R signalling is mediated by HIF-1α. (**A**) BRIN-BD11 cells were treated as indicated for 18 hours and Hif1α protein levels were determined by immunoblot analysis. (**B**) After treatment with Exendin-4 for 18 hours HIF-1α mRNA expression was evaluated by qRT-PCR. (**C**) BRIN-BD11 cells were pre-incubated with either vehicle, 1 µM Torin 1 or 50 µM LY294002 for 30 min as indicated, followed by addition (or not) of 50 nM Exendin-4 for 18 hours. Immunoblot shows Hif1α protein expression and phosphorylation levels of mTOR at serine 2448. (**D**–**J**) BRIN-BD11 cells were transfected with either HIF-1α specific siRNAs (*siHIF1a*) or non-targeting siRNAs (*siControl*) for 24 h, and subsequently exposed to 50 nM Exendin-4 (or not) for 18 h. (**D**) Exendin-4’s ability to promote protein levels of Pdh and Pfkp was significantly blunted in cells transfected with *siHIF1a* as compared to *siControl* transfected cells. (**E**) At the mRNA level, *siHIF1a* transfection caused a reduction of approximately 80% in HIF-1α expression. (**F**) mRNA expression of glycolytic enzymes was determined by qRT-PCR analysis. (**G**–**J**) Determination of glucose consumption, 2-NBDG uptake, SSC-A and FSC-A following HIF-1α knockdown. Data are mean ± SEM, n ≥ 3; n.s. = non-significant; *P < 0.05; **P < 0.01; ***P < 0.001; ****P < 0.001.

HIF-1 targets expression of glycolytic enzymes^28^; furthermore, GLP-1 has been shown to promote HIF-1α translation *via* induction of mTOR in mouse islets^16^. We hypothesized that HIF-1α mediates Exendin-4 effects on glycolysis in our model. Immunoblot analysis confirmed that Exendin-4 treatment induced HIF-1α protein levels similar to those achieved with Forskolin (Fig. 6A). Gene expression analysis revealed no changes in *HIF-1α* mRNA, suggesting that GLP-1R signalling induces HIF-1α protein by a post-transcriptional mechanism (Fig. 6B), consistent with previous observations^16^. Next, we assessed how HIF-1α levels are modulated by the activity of PI3K/mTOR axis in this setting. Inhibition of either PI3K or mTOR abolished accumulation of HIF-1α triggered by Exendin-4, suggesting that such event occurs downstream of mTOR (Fig. 6C). In addition, the two inhibitors caused HIF-1α protein to drop further below basal levels, indicating that mTOR pathway controls HIF-1α additionally in unstimulated conditions. Moreover, phosphorylation of mTOR at S2448 was enhanced by Exendin-4, confirming that GLP-1R signalling increases the activation state of mTOR, as previously demonstrated^16, 29, 30^. Knockdown of HIF-1α expression by siRNA transfection resulted in an approximate reduction in HIF-1α protein levels by 60% (Fig. 6D) and mRNA levels by 80% (Fig. 6E). We then sought to determine how HIF-1α knockdown influences the effects of Exendin-4 on glycolytic gene expression. Induction of Pdh and Pfkp by Exendin-4 was significantly blunted by HIF-1α knockdown (Fig. 6D). Protein levels of Pkm1, Pkm2, Hexokinase 1 and Hexokinase 2 were not affected (Fig. S4). Furthermore, depletion of HIF-1α caused a reduction in mRNA expression of glycolytic genes investigated, although only *Pgam2* was statistically significant (Fig. 6F). This was accompanied by a reduction in glucose consumption and uptake in response to Exendin-4 (Fig. 6G and H, respectively), and also blocked the effects of Exendin-4 on cellular complexity and size (Fig. 6I and J, respectively). These findings indicate that HIF-1α was necessary for the observed effects of GLP-1R signalling on glucose metabolism.

## Discussion

Treatment of T2D patients with GLP-1 analogues results in various beneficial effects, including improvement in β-cell function and glucose homeostasis^7^. Yet, molecular mechanisms underlining GLP-1 mediated stimulation of insulin secretion are still not fully characterized. GLP-1-induced insulin secretion is believed to rely on the direct effects of cAMP signalling on ATP sensitive K^+^ channels and Ca^2+^ handling^14^. Such mechanisms occur very rapidly, within 5–15 min upon receptor binding, indicating that the actions are not mediated by altered gene expression, but likely by phosphorylation of protein components of the secretory machinery^13^. GLP-1 is known to acutely induce glucose competence in β-cells, both in isolated islets and in human subjects^31, 32^. Data presented herein support the notion that prolonged exposure to GLP-1R stimulation can also induce glucose competence by a secondary mechanism involving up-regulation of glycolytic enzymes, and consequently glycolytic flux, arguably the most important stimulus-secretion coupling factor generating pathway in β-cells. Other lines of evidence also suggest that GLP-1 may have additional effects other than the well-known acute insulin secretion stimulation. For instance, GLP-1 analogues promoted persistent improvement of β-cell function four weeks after treatment interruption, both in rodents and humans^21, 33^. Although this concept has been recently challenged with the publication of clinical trials showing that Liraglutide^34^ and Exendin-4^35^ enhancement of β-cell function was lost after treatment cessation. However, it may be that the effect cannot be sustained for several weeks after treatment interruption.

Here we provide evidence that prolonged GLP-1R activation induces a robust metabolic adaptation in β-cells. In our model, cells primed with Exendin-4 presented higher glycolytic rates when returned to culture media in the absence of GLP-1R stimulation. This is concluded from evidence provided by glucose consumption, 2-NBDG uptake and lactate production measurements as well as ECAR and OCR profiles (Figs 1 and 2). BRIN-BD11 cells were used throughout this study for their well characterised responses to multiple stimuli, cell culture based convenience and their established intact response to GLP-1R activation both in terms of cAMP and resulting stimulated insulin secretion^36, 37^. We also validated findings such as Exendin-4 effects on glucose consumption and insulin secretion in primary isolated mouse islets. We believe that the increase in glucose metabolism occurs *via* up-regulation of glycolytic enzymes, since the observed metabolic effects were accompanied by significant upregulation in mRNA and protein expression of critical glycolytic genes (Fig. 3). In addition, our data also showed that acute Exendin-4 stimulation does not elicit change in metabolic flux (Fig. 2). Corroborating this hypothesis, HIF-1α, a transcriptional factor known to target glycolytic genes, accumulates under GLP-1R stimulation. Furthermore, HIF-1α depletion impacted glucose consumption and uptake, as well as glycolytic gene expression in the same setting (Fig. 6). The dataset presented here also indicate that the PI3K/mTOR pathway plays a crucial role in mediating GLP-1R signalling effects on energy metabolism. This is evident from experiments describing the effects of specific PI3K and mTOR inhibitors, which completely abrogated the metabolic actions of Exendin-4 (Fig. 5). Accordingly, HIF-1α accumulation was also abrogated upon PI3K/mTOR inhibition, further demonstrating its importance for the observed metabolic adaptations (Fig. 6). We and others have demonstrated that GLP-1R signalling elevated ATP levels^38, 39^, strengthening the hypothesis that the incretin hormone may promote β-cell function through regulation of energy metabolism, especially considering the central role that ATP generation and an increase in ATP/ADP ratio play during GSIS. Indeed, in our model, prolonged GLP-1R stimulation promoted a sustained increase in insulin secretion after Exendin-4 removal; this was observed both in BRIN-BD11 cells and murine islets (Fig. 1). We suspect that metabolic/bioenergetic reprograming, observed in this study, could also mediate short-term maintenance of β-cell function upon withdrawal of GLP-1 analogues, as documented in humans and rodents by others^21, 33^. Clearly though, such sustained effect will be completely lost after a few weeks of treatment interruption, as recent clinical evidence suggests^34, 35^.

Previously, it has been reported that acute exposure to GLP-1 did not significantly affect the metabolome^40^, nor bioenergetics of rodent β-cells^15^. Gheni *et al*. demonstrated that exposure to GLP-1 for 30 min did not induce changes to glycolytic and TCA cycle intermediates in MIN6-K8 cells^40^. A short-term (90 min) stimulation with GLP-1 did not cause changes in glucose and lipid oxidation in rodent islets^15^, although, the same study reported a significant increase in glucose utilization, which perhaps has not been given sufficient attention. Accordingly, our data also showed that acute GLP-1R stimulation did not cause changes to glucose metabolism. Hence, we speculate that the increase in glucose utilization observed by Peyot *et al*.^15^ reflects a different mechanism, reported recently, in which GLP-1 induces rapid post-translational activation of glucokinase^41, 42^. Data presented herein support a novel mechanism dependent on metabolic adaptations observed after a longer time period, which is consistent with previously identified delayed phase of gene expression changes mediated *via* HIF-1α^16^.

Interestingly, a recent study by the Mandrup laboratory has shown that exposure to high glucose (25 mM) elicits a biphasic response in INS-1E cells^43^. In that study, the glucose-sensing transcription factor carbohydrate response element binding protein (ChREBP) has been shown to initiate a regulatory program driving an early transcriptional activation of metabolic genes, which is followed by a second wave of transcriptional regulation wherein cell-cycle genes are activated and β-cell identity genes are repressed. In our model, β-cells were exposed to Exendin-4 and high glucose concomitantly. Therefore, the metabolic adaptations observed here probably occurred in the background of the recently identified glucose-induced transcriptional program. This suggests that ChREBP and downstream associated transcription factors might also play a role in the observed metabolic shift. This hypothesis is also supported by our HIF-1α knockdown experiments, in which GLP-1R signalling-induced gene expression and metabolic adaptations were only partially perturbed, pointing to a possible involvement of additional transcriptional factors. Future studies aiming at identifying global changes in gene regulation in response to prolonged exposure to GLP-1R stimulation can determine with greater detail the remaining players in the herein identified metabolic reprograming process.

**Figure 7:**
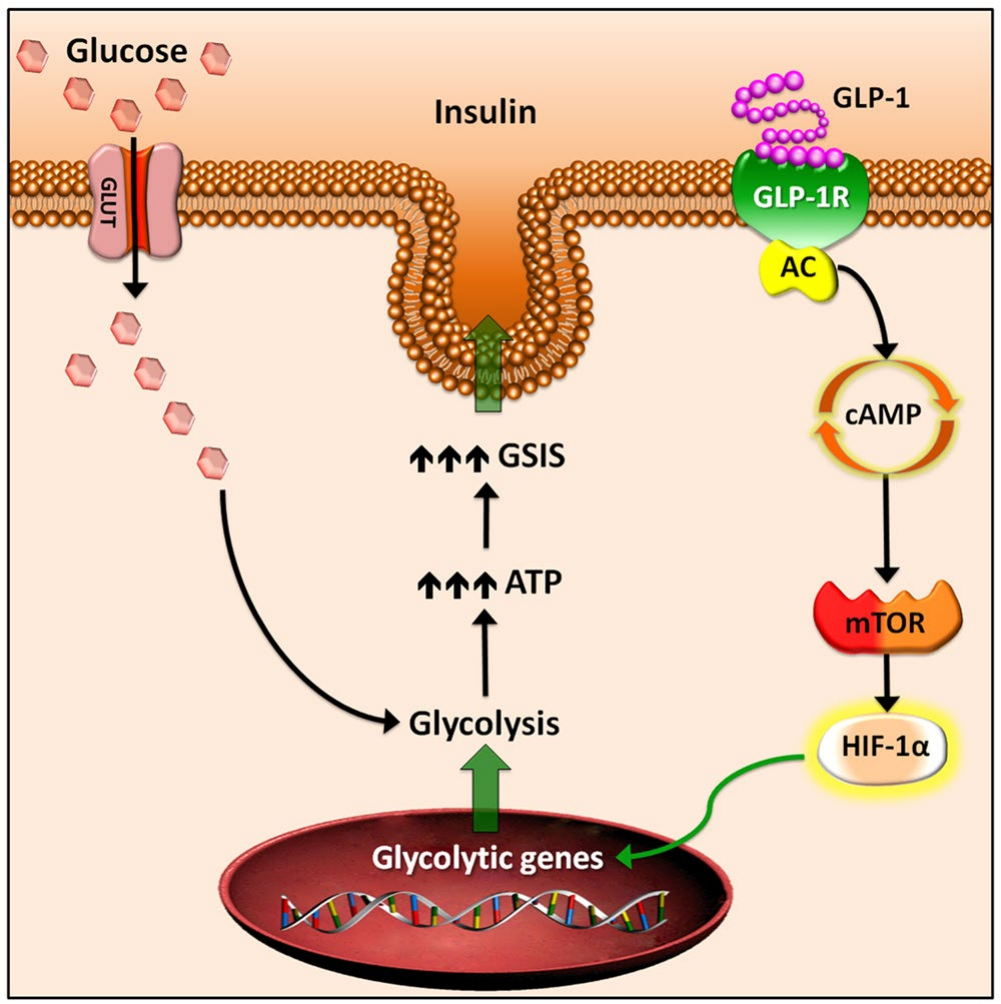
GLP-1R signalling promotes β-cell glucose metabolism *via* mTOR-dependent HIF-1α activation. Upon GLP-1 binding, the G-protein coupled GLP-1R receptor activates adenylyl cyclase (AC), increasing intracellular levels of cAMP. Signalling mediated by cAMP promotes the mTOR pathway in β-cells, which, in turn, induces translational activation of HIF-1α. In the nucleus, HIF-1α drives transcriptional up-regulation of glycolytic genes. Increased pool of glycolytic enzymes in the cytosol allows for an elevated glycolytic flux and glycolytic capacity, resulting in faster generation of ATP and enhanced glucose stimulated insulin secretion (GSIS).

Our study describes a novel action of GLP-1 signalling in β-cells, which stimulates metabolic/bioenergetic reprograming resulting in a high glycolytic/high ATP generating phenotype, as detailed in (Fig. 7). Such metabolic reprograming likely accounts for a major part of the mechanism(s) underlying GLP-1 stimulated insulin secretion. Our study provides evidence for an important mechanism by which GLP-1 analogues improve glucose homeostasis in T2D patients, unveiling new aspects that could be useful for developing new therapeutic strategies.

## Methods

### Animals

Male C57BL/6J mice at 5–7 weeks of age were used for the purpose of pancreatic islets isolation. All animals were cared according to the guidelines for use and care of laboratory animals^44^. The study was approved by Curtin Ethics Committee for the use of animals in research (AEC-2014-27).

### Reagents

All chemicals were purchased from Sigma-Aldrich, unless indicated otherwise. Rabbit polyclonal antibody against HIF-1α was obtained from (Novus Biologicals, Littleton, CO, USA, #NB100–449), while phosphorylated mTOR S2448, β-actin, α-Tubulin, as well as Glycolysis Antibody Sampler Kit to detect Pfkp, Pkm2, Pkm 1/2, Hexokinase 1, Hexokinase 2 and Pdh were all obtained from (Cell Signaling Technology, Beverly, MA, USA, #2971, #4970, #2148, #8337 respectively). Secondary antibodies consisted of horseradish peroxidase conjugated goat anti-rabbit IgG (Agilent’s Dako, Glostrup, Denmark). Torin 1, LY294002 and Forskolin were purchased from (ApexBio, Houston, TX, USA). ON-TARGETplus Rat Hif1a siRNA – SMARTpool (siHif1a) and Non-targeting Pool siRNA (siControl) were purchased from (Dharmacon, Lafayette, CO, USA - L-091718-02 and D-001810-10-05, respectively).

### Cell culture

Clonal insulin-secreting BRIN-BD11 cells were maintained in RPMI 1640 medium containing 11.1 mM glucose, supplemented with 10% foetal bovine serum (FBS), 100 U/ml penicillin and 0.1 mg/ml streptomycin, pH 7.4, as described previously^45^. Cells were maintained in T75 sterile tissue culture flasks at 37 °C in a humidified atmosphere of 5% CO_2_ and 95% air.

### Islet isolation

Islets were isolated by bile duct perfusion followed by collagenase P digestion as described previously^46^. After purification, islets were cultured overnight at 37 °C in a humidified atmosphere of 95% air and 5% CO_2_ in RPMI 1640 medium supplemented with 10% FBS, 100 units/ml penicillin and 0.1 mg/ml streptomycin. Islets were subsequently hand-picked and placed into individual micro centrifuge tubes for glucose consumption and insulin secretion assays.

### Glucose consumption, lactate production, insulin secretion and total ATP content assays

After pre-conditioning in the presence or absence of 50 nM Exendin-4 for 18 hours, BRIN-BD11 cells or isolated mouse islets were cultured in RPMI containing 20 mM of glucose in the absence of Exendin-4 for additional 24 hours. Tissue culture media supernatants from the final 24 hours of culture were collected and assayed by Amplex Red Glucose/Glucose Oxidase Assay Kit (Life Technologies, Gaithersburg, MD, USA), Lactate Assay kit (Sigma-Aldrich) and Rat Ultrasensitive Insulin ELISA (Mercodia, Uppsala, Sweden) to measure total glucose, lactate and insulin respectively. Cells were lysed with RIPA buffer (Astral Scientific, Sydney, Australia) and total DNA quantified using Quanti-iT PicoGreen (Life Technologies, Gaithersburg, MD, USA). Results were reported as nmole of glucose and lactate, or pg of insulin. Results were normalized by total DNA or number of islets. For total ATP content measurements, cells were lysed with CellTiter-Glo® Reagent (Promega Corporation, Madison, WI, USA) and total ATP determined. All measurements were performed according to manufacturer’s instructions.

### Glucose uptake assay

Glucose uptake was monitored with the fluorescent deoxyglucose analogue 2-(N-(7-nitrobenz-2-oxa-1,3-diazol-4-yl)amino)-2-deoxyglucose (2-NBDG) (Thermo Fisher Scientific, San Jose, CA, USA)^47^. After pre-conditioning, cells were washed and incubated in glucose-free DMEM medium containing 20 μM 2-NBDG for 30 min at 37 °C. Unlabelled controls were generated by incubating cells as described above, however in the absence of 2-NBDG. Cells were recovered by trypsinization and resuspended in 100 μL of PBS containing 1 μg/mL propidium iodide (PI). Ten thousand cells were evaluated by FACS LSR Fortessa flow cytometer (BD Biosciences, Heidelberg, Germany) and data were analysed in the FlowLogic FCS analysis software (Inivai Technologies, Melbourne, Australia). Median 2-NBDG, side scatter (SSC) and forward scatter (FSC) fluorescence were obtained after gating for PI negative cells.

### Extracellular flux analysis

Bioenergetic parameters were determined using the XF^e^96 Extracellular Flux Analyzer (Seahorse Bioscience, North Billerica, MA). Cells were seeded into specialized 96-well plates at a density of 10^4^ cells/well and allowed to adhere overnight. After pre-conditioning in the presence or absence of 50 nM Exendin-4 for additional 18 hours, culture medium was changed to serum-free DMEM containing 1 mM sodium pyruvate, without glucose and sodium bicarbonate. Plates were then incubated for one hour at 37 °C in a CO_2_-free atmosphere. Basal oxygen consumption rate (OCR) and extracellular acidification rate (ECAR) were determined. Next, OCR and ECAR profiles in response to injection of glucose (25 mM), oligomycin (2 µM), carbonyl cyanide-*4*-(trifluoromethoxy) phenylhydrazone (FCCP) (0.3 µM), and a combination of antimycin A (1 µM) and rotenone (1 µM) were evaluated. OCR and ECAR were measured using five 2 min cycles of mix and measurement following each injection. Normalization was performed by assessing total DNA using Quanti-iT PicoGreen (Life Technologies, Gaithersburg, MD, USA). Data analyses and calculations were performed as previously described^48^.

### Quantitative RT-PCR

RNA was extracted from cell lysates using RNeasy Mini Kit (Qiagen, Chatsworth, CA, USA) and was reverse-transcribed using QuantiTect Reverse Transcription Kit (Qiagen, Chatsworth, CA, USA). Gene expression levels were quantified by qPCR using QuantiFast SYBR Green PCR Kit (Qiagen, Chatsworth, CA, USA) and reactions were performed on a Viia^TM^ 7 real time PCR system (Life Technologies, Gaithersburg, MD, USA). Gene-specific amplification was achieved using RT^2^ qPCR Primer Assays (Qiagen, Chatsworth, CA, USA). Catalogue numbers for each primer assays are presented in (Supplementary Table 1). Relative gene expression was determined by normalizing the expression of each target gene to β-actin.

### Immunoblot analysis

HIF-1α protein is rapidly degraded by the ubiquitin-proteasome system under normoxic conditions^49^. In order to avoid further HIF-1α degradation during whole cell lysates preparation, cells were lysed directly with NuPAGE® LDS Sample Buffer (1X) (Thermo Fisher Scientific, San Jose, CA, USA) in the presence of protease and phosphatase inhibitors cocktail (Cell Signaling Technology, Danvers, MA, USA), similarly to ref. 50. Protein extracts were separated by electrophoresis on 4–12% NuPAGE Bis-Tris Mini Gel and then transferred onto nitrocellulose membranes using iBlot transfer stacks (Life Technologies, Gaithersburg, MD, USA). Membranes were probed with various primary antibodies overnight at 4 °C. All washes and secondary antibody incubations were performed using the SNAP i.d. quick immunoblot vacuum system (Millipore, Billerica, MA, USA). Bands were developed using Clarity Western ECL substrate (Bio-Rad Laboratories, Hercules, CA, USA). Visualization and quantitative densitometry analysis were performed with Molecular Imager® Gel Doc™ XR System v5.2.1 (Bio-Rad Laboratories, Hercules, CA, USA).

### Statistical analysis

Variables are reported as mean ± S.E.M. Differences across groups were tested using ANOVA with a Tukey post hoc test when more than two experimental groups were analysed and using Student’s t-test when only two experimental groups were compared. Statistical significance was set at P < 0.05 (two-tailed). All analyses were performed using GraphPad Prism software v. 6.0.

## Electronic supplementary material

Supplementary Information
