# Supplementary material for: GLP-1 receptor signalling promotes β-cell glucose metabolism via mTOR-dependent HIF-1α activation
Source: Sci Rep. 2017 Jun 1;7:2661. doi: 10.1038/s41598-017-02838-2 (PMC5454020; doi:10.1038/s41598-017-02838-2)
Supplement: Supplementary file 1 — Supplementary Information [file 41598_2017_2838_MOESM1_ESM.pdf]

GLP-1 receptor signalling promotes β-cell glucose metabolism *via* mTOR-dependent HIF-1α activation

Rodrigo Carlessi, Younan Chen, Jordan Rowlands, Vinicius F. Cruzat, Kevin N. Keane, Lauren Egan, Cyril Mamotte, Rebecca Stokes, Jenny E. Gunton, Paulo Ivo Homem de Bittencourt Jr., Philip Newsholme

Supplementary Information

Supplementary Table 1. List of genes assessed by qRT-PCR and respective RT^2 qPCR Primer Assay catalogue numbers.

Gene Symbol	Refseq #	Qiagen Catalogue Number
Pklr	NM_012624	PPR50315
Pgam2	NM_017328	PPR44447
Gpi	NM_207592	PPR52501
Pfkp	NM_206847	PPR59714
Aldoa	NM_012495	PPR42582
Ldha	NM_017025	PPR56603
Actb	NM_031144	PPR06570

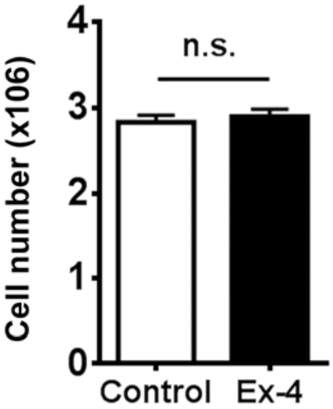

S1. **18h of Ex-4 treatment does not induce BRIN-BD11 cell proliferation.** BRIN-BD11 cells were seeded in 6 well plates at a density of 10^6 cells per well and allowed to grow overnight. Cells were then treated in the presence or absence of 50 nM Exendin-4 for additional 18h in media containing 20 mM of glucose. At the end of incubation time cells were manually counted using a Neubauer chamber. Data are mean ± SEM, n = 6; n.s. = non-significant.

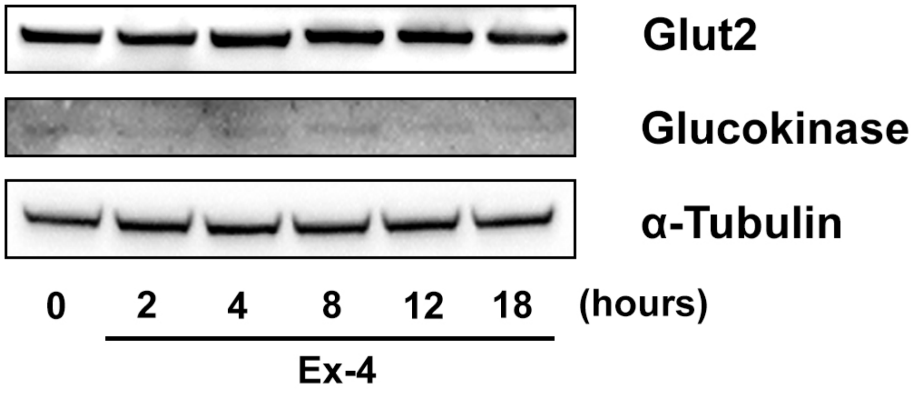

S2. Protein levels of Glut2 and Glucokinase are unaffected by prolonged exposure to Exendin-4. BRIN-BD11 cells were treated with 50 nM Exendin-4 for 2, 4, 8, 12 and 18 hours followed by immunoblot analysis of Glut2 and Glucokinase. Results are representative of at least three independent experiments.

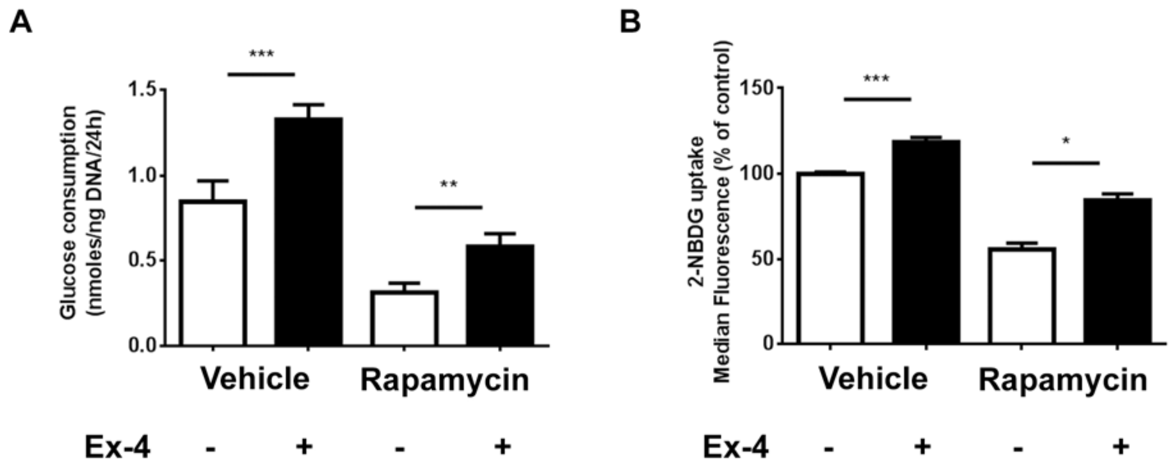

S3. **Exendin-4 conserves stimulatory activity in the presence of mTORC1 inhibitor, Rapamycin.** (A) BRIN-BD11 cells were pre-incubated with either vehicle or 100 nM Rapamycin for 30 min, followed by addition (or not) of 50 nM Exendin-4 for 18 hours, as indicated. Then, media was changed to RPMI containing 20 mM of glucose for additional 24 hours in the absence of Exendin-4 and glucose consumption was determined. (B) Cells were treated similarly to (A), but instead of the additional 24 hours incubation time, 2-NBDG

uptake was performed immediately following the 18 hours incubation period as described in Methods. Data represent mean ± SEM, n = 3; *P < 0.05; **P < 0.01; ***P < 0.001.

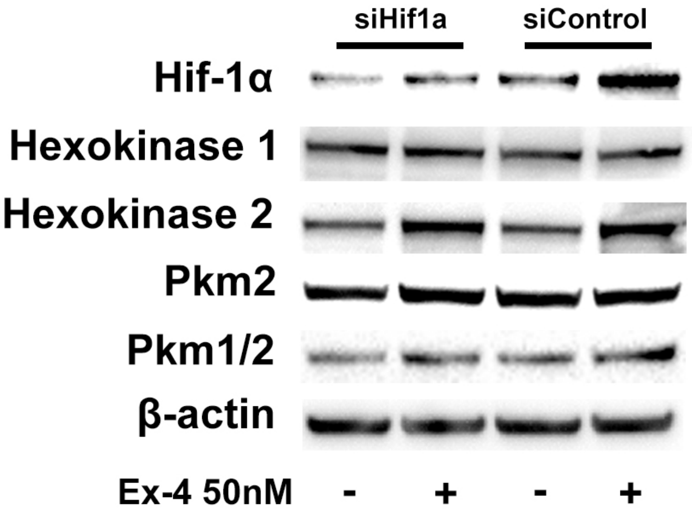

S4. There is no evidence of an effect on protein levels of Hexokinase 1, Hexokinase 2, Pyruvate Kinase M 1 and M2 in response to HIF-1α knockdown by siRNA transfection. BRIN-BD11 cells were transfected with either HIF-1α specific siRNAs (*siHIF1a*) or non-targeting siRNAs (*siControl*) for 24h, and subsequently exposed to 50 nM Exendin-4 (or not) for 18h. Results are representative of at least three independent experiments.
